# GTRpmix: A Linked General Time-Reversible Model for Profile Mixture Models

**DOI:** 10.1093/molbev/msae174

**Published:** 2024-08-19

**Authors:** Hector Banos, Thomas K F Wong, Justin Daneau, Edward Susko, Bui Quang Minh, Robert Lanfear, Matthew W Brown, Laura Eme, Andrew J Roger

**Affiliations:** Department of Mathematics, California State University San Bernardino, San Bernardino, CA, USA; Department of Biochemistry and Molecular Biology, Faculty of Medicine, Dalhousie University, Halifax, NS, Canada; School of Computing, College of Engineering and Computing and Cybernetics, Australian National University, Canberra, ACT 2600, Australia; Ecology and Evolution, Research School of Biology, College of Science, Australian National University, Canberra, ACT 2600, Australia; Department of Biochemistry and Molecular Biology, Faculty of Medicine, Dalhousie University, Halifax, NS, Canada; Department of Mathematics and Statistics, Faculty of Science, Dalhousie University, Halifax, NS, Canada; School of Computing, College of Engineering and Computing and Cybernetics, Australian National University, Canberra, ACT 2600, Australia; Ecology and Evolution, Research School of Biology, College of Science, Australian National University, Canberra, ACT 2600, Australia; Department of Biological Sciences, Mississippi State University, Mississippi State, MS, USA; Laboratoire d’Ecologie, systématique et Evolution, Université Paris-Saclay, Gif-sur-Yvette, France; Department of Biochemistry and Molecular Biology, Faculty of Medicine, Dalhousie University, Halifax, NS, Canada

**Keywords:** frequency profile mixtures, phylogenetics, maximum likelihood, model of sequence evolution, general time reversible model

## Abstract

Profile mixture models capture distinct biochemical constraints on the amino acid substitution process at different sites in proteins. These models feature a mixture of time-reversible models with a common matrix of exchangeabilities and distinct sets of equilibrium amino acid frequencies known as profiles. Combining the exchangeability matrix with each profile generates the matrix of instantaneous rates of amino acid exchange for that profile. Currently, empirically estimated exchangeability matrices (e.g. the LG matrix) are widely used for phylogenetic inference under profile mixture models. However, these were estimated using a single profile and are unlikely optimal for profile mixture models. Here, we describe the GTRpmix model that allows maximum likelihood estimation of a common exchangeability matrix under any profile mixture model. We show that exchangeability matrices estimated under profile mixture models differ from the LG matrix, dramatically improving model fit and topological estimation accuracy for empirical test cases. Because the GTRpmix model is computationally expensive, we provide two exchangeability matrices estimated from large concatenated phylogenomic-supermatrices to be used for phylogenetic analyses. One, called Eukaryotic Linked Mixture (ELM), is designed for phylogenetic analysis of proteins encoded by nuclear genomes of eukaryotes, and the other, Eukaryotic and Archaeal Linked mixture (EAL), for reconstructing relationships between eukaryotes and Archaea. These matrices, combined with profile mixture models, fit data better and have improved topology estimation relative to the LG matrix combined with the same mixture models. Starting with version 2.3.1, IQ-TREE2 allows users to estimate linked exchangeabilities (i.e. amino acid exchange rates) under profile mixture models.

## Introduction

Models of amino acid substitution are of key importance to probabilistic molecular phylogenetic analyses of protein sequences. Typically, the amino acid substitution process is modeled via a site-independent time-reversible Markov process on a tree. The parameters of this model include a set of fixed equilibrium frequencies of the amino acids (referred to in this work as a profile) and a fixed matrix of amino acid exchange rates—exchangeabilities—throughout the tree. The exchangeability matrix accounts for some biological, chemical, and physical amino acid properties and when combined with the equilibrium frequencies of the amino acids, it describes the instantaneous rates of interchange between each pair of amino acids.

In most phylogenetic analyses the exchangeability matrix used is chosen from a set of fixed empirically estimated matrices. The first empirically estimated exchangeabilities were derived from the Dayhoff (Dayhoff et al. 1978) and Jones–Taylor–Thornton (JTT) (Jones et al. 1992) matrices that were obtained by counting the substitutions between each amino acid pair using ancestral sequence reconstruction and a parsimony-based approach to analyze databases of multiple alignments along with their estimated phylogenies. Subsequently, a maximum likelihood approach was used to improve exchangeability estimation leading to the development of the “Whelan and Goldman” (WAG) model (Whelan and Goldman 2001). Le and Gascuel expanded this approach (Le and Gascuel 2008) by considering larger data sets and by incorporating heterogeneity of rates across sites in the likelihood computation via a site-rate partition model. The resulting matrix, known as the “Le and Gascuel” (LG) matrix, is currently very widely used for phylogenetic inference based on protein sequences. Expanding on these, Minh et al. (2021) introduced QMaker, a maximum likelihood method to estimate an exchangeability matrix from a large protein data set consisting of multiple independent sequence alignments. The authors used QMaker to estimate a number of additional matrices to be used for phylogenetic analyses of specific taxonomic groups (e.g. Q.bird, Q.insect, and Q.plant). Other matrices have been developed to fit proteins encoded on certain organellar genomes (e.g. cpREV Adachi et al. 2000) or particular gene families (e.g. rtREV Dimmic et al. 2002).

All of the foregoing exchangeability matrices were obtained assuming that all sites evolve according to the same process and share a single set of equilibrium amino acid frequencies (a single profile). However, because of different functional constraints and structural microenvironments within proteins, there are distinct ranges of admissible amino acids at sites (Pál et al. 2006; Goldstein 2008; Franzosa and Xia 2009). Profile mixture models, such as the C10–C60 series and the UDM series (Si Quang et al. 2008; Wang et al. 2008, 2014; Schrempf et al. 2020), were designed to account for this heterogeneity of preferred amino acids across sites. These models are mixtures of time-reversible Markov models, but they assume a common exchangeability matrix and distinct profiles of stationary frequencies.

Exchangeabilities and amino acid profiles capture, in different ways, similar properties of the amino acid replacement processes across sites. Ideally, one would want to separate the properties that are captured by exchangeabilities versus profiles. However, this is nontrivial since such properties depend on features like the structural context of sites in proteins, information that is absent from the data used for analysis (see, for example, Spielman and Wilke 2015). In current site-homogeneous approaches to the estimation of exchangeabilities, site-specific amino acid preferences are not explicitly modeled, so exchangeabilities indirectly capture some of these site-specific signals as average effects. It would be preferable if the profiles modeled site-specific selective constraints and the exchangeabilities modeled alignment-wide aspects of the substitution process (e.g. mutational processes and genetic code effects). Unfortunately, it is doubtful that these two aspects of substitution processes can be completely disentangled. Nevertheless, it seems clear that unless profiles are included when estimating exchangeabilities, estimates of the latter will reflect site-specific properties to a considerable degree. This highlights the importance of re-estimating exchangeabilities in the context of mixture models to avoid redundancy between the signals captured between profiles and exchangeabilities.

Estimation of a single exchangeability matrix, within a profile mixture setting, has been explored in a Bayesian context by Lartillot and Philippe (2004) through the development of various versions of the CAT model of Phylobayes (Lartillot et al. 2013). The CAT-GTR model uses Markov chain Monte Carlo techniques to jointly infer frequency vectors, exchangeabilities, the affiliations of each site to a given frequency vector, the rates at each site, the branch lengths, and the tree topology. However, in practice, convergence may not be achieved in large data sets with many sites and taxa in its current implementation (Lartillot et al. 2013). In the maximum likelihood framework, Wong and colleagues developed MAST (Wong et al. 2024), an extension of IQ-TREE2 that, among other things, allows the user to estimate a mixture model with various options for linking and unlinking exchangeability matrices and amino acid profiles, in conjunction with mixtures of tree topologies. While this implementation can be very useful in many contexts, it is not practical for profile mixture models with many profiles because, for each profile, 189 exchangeability parameters need to be estimated. For commonly used models with 40 to 60 profiles (e.g. C40 or C60) or more (e.g. UDM64, UDM256, etc.), this corresponds to >>7,500 estimated parameters. These models would require complex and computationally expensive optimization and will potentially be susceptible to problems associated with local optima, over-parameterization, and identifiability.

Here, we describe the implementation of a General Time-Reversible model via maximum likelihood estimation in IQ-TREE2 for use with profile mixture models. This GTR model (denoted as GTR20 in IQ-TREE2) has a single set of optimizable exchangeability parameters shared (“linked”) over all classes of the profile mixture. By simulation, we show that our implementation accurately estimates exchangeability parameters and that it can improve tree topology estimation accuracy. Additionally, we show that the estimation of exchangeabilities under a profile mixture model provides a much-improved fit on a well-known empirical data set than the profile mixture model with LG exchangeabilities.

Since the estimation of exchangeabilities can be computationally expensive and requires large data sets for accurate parameter estimates, we provide two matrices estimated from large concatenated supermatrices under the GTR-C60 profile mixture model to be used as fixed matrices for phylogenetic analyses. One of these, called eukaryotic linked mixture (ELM), is tailored for phylogenetic analyses of proteins encoded by eukaryotic nuclear genes, and the other, eukaryotic and archaeal linked (EAL), is for reconstructing relationships between eukaryotes and Archaea. We show, via three well-known empirical data sets, that these matrices have better fit and topological accuracy than the LG matrix when both are combined with C60. Additionally, we show that these matrices perform well with different sets of profiles.

## Profile Mixture Models and Exchangeability Optimization

The general time-reversible model (GTR) (Tavaré 1986) is a Markov process where, for a profile $\pi =(\pi_{1},\pi_{2},\ldots ,\pi_{20})$, with $\sum_{a=1}^{20}\pi_{a}=1$, and a matrix *Q* of instantaneous rates of change between amino acids, diag$(\pi )Q=Q^{T}$diag(π). Because of this, one can parameterize *Q* via a non-negative symmetric matrix *S* known as the exchangeability matrix. Specifically, given an exchangeability matrix $S=\{s_{i,j}{\}}_{i,j=1}^{20}$ and a profile π, the entries of the time-reversible instantaneous rate matrix $Q_{\pi }=\{q_{i,j}{\}}_{i,j=1}^{20}$ associated to π are defined as

1.
$q_{ij}=s_{ij}\pi_{j}$ , for i≠j and $q_{ii}=-\sum_{\;j=1,i\neq j}^{20}q_{ij}$, otherwise.
2. multiplying all entries by $(-\sum_{i=1}^{20}q_{ii}\pi_{i}{)}^{-1}$, so that branch lengths are interpretable as expected number of substitutions per site.

All entries of $Q_{\pi }$ are non-negative, row sums are 0, $\pi Q_{\pi }=0$, and diag$(\pi )Q_{\pi }$ is symmetric. For any given π and any c>0, exchangeability matrices *S* and *cS* yield the same instantaneous rate matrix $Q_{\pi }$, and thus produce the same site-pattern probabilities. Therefore, we constrain one entry to be equal to 1 resulting in 189 free parameters from the exchangeability matrix.

Commonly used profile mixture models are, usually, mixtures of time-reversible models with a common exchangeability matrix *S*. Specifically, site profiles $\pi_{c}$ are selected independently with probability $w_{c}$ and, independently of these, rates for sites, $r_{k}$, are chosen with probability $d_{k}$. Given the rates and site profile for a site *p*, the evolutionary model is a GTR process with exchangeability matrix *S* along a tree *T*. Let $P(x_{p}\;|\;T,S,\pi_{c},r_{k})$ denote the conditional probability of site pattern $x_{p}$ given its site rate, $r_{k}$, and site profile, $\pi_{c}$. Because the rates and site profiles are unobserved, the likelihood contribution under the model for the site is the marginal probability of the site pattern,


(1) $$L(S,T,\{(\pi_{c},w_{c})\},\{(r_{k},d_{k})\};x_{p})=\sum_{c=1}^{C}w_{c}\sum_{k=1}^{K}d_{k}P(x_{p}\;|\;T,S,\pi_{c},r_{k}),$$

were $\{\pi_{c}{\}}_{c=1}^{C}$ is a collection of *C* profiles with corresponding positive weights $\{w_{c}{\}}_{c=1}^{C}$ summing to one, $\{r_{k}{\}}_{k=1}^{K}$ is a collection of *K* non-negative scalar rate parameters with corresponding positive rate weights $\{d_{k}{\}}_{k=1}^{K}$ also summing to one, and $\sum_{k=1}^{K}d_{k}r_{k}=1$.

To reduce the complexity and computational cost of profile mixture models, fixed profiles are typically used for tree estimation (Si Quang et al. 2008; Wang et al. 2008; Schrempf et al. 2020; Tice et al. 2021; Eme et al. 2023). In these cases, the only additional parameters coming from the profile mixture are the weights of the profile, giving C−1 additional free parameters from the weights, where *C* is the number of profiles. Different sets of profiles have been estimated via databases of alignments. For example, Si Quang et al. (2008) introduced the widely used sets of profiles known as C10, C20, C30, C40, C50, and C60 (generically referred to as CXX). These sets of profiles were estimated under uniform exchangeabilities (referred to as POISSON exchangeabilities). In each of these, the number next to the “C” denotes the number of profiles in the set. Other sets of profiles include the more recently introduced UDM models (Schrempf et al. 2020), with sets of profiles ranging from 4, 8, 16, and up to 4,096 classes.

For rates across sites, it is common to use a discretized approximation to the gamma distribution with shape parameter *α* and mean 1 (Yang 1994). For these distributions, all rates have an equal probability of occurrence and are continuous functions $r_{k}(\alpha )$ of *α*. The shape parameter *α* adds only one free parameter to a profile mixture model. The discretized gamma distribution is commonly discretized into 4-rate classes and is denoted G4.

In most of our analyses with the C-series models below, we estimate the weights of the mixture; the default of IQ-TREE is to use empirical weights (weights obtained during the estimation of the original empirical profile frequencies, rather than being re-estimated for the data at hand) unless a “+F” component is included. In a similar fashion, unless clearly stated, we also jointly estimate the shape parameter *α*. Lastly, exchangeabilities are also jointly estimated, unless clearly stated to be fixed a priori to previously estimated exchangeabilities (for example, to LG or POISSON).

Given an MSA with *n* sites and a tree *T*, we estimate the exchangeabilities by maximizing the log-likelihood across all sites


(2) $$\ell (S)=\sum_{\;p=1}^{n}\log\;L(S,T,\{(\pi_{c},w_{c})\},\{(r_{k},d_{k})\};x_{p}).$$

To do this, we arbitrarily fix the exchangeability between *Y* and *V* (corresponding to the entry $s_{19,20}$ of *S*) to 1, and we then estimate the 189 remaining exchangeabilities using the BFGS-algorithm (Fletcher 1987), a well-known iterative optimization method. By default, the algorithm is initialized with all 189 exchangeabilities equal to one, with the option to specify any other initial exchangeabilities. In its current implementation, other parameters of the profile mixture can be jointly estimated using IQ-TREE2’s routines. For example, one can simultaneously estimate the tree topology, branch lengths, rates (not necessarily from a discretized gamma), weights of fixed profiles, and exchangeabilities (or any subset of this list).

We compare exchangeabilities *S* and $S^{\prime }$ via their associated rate matrices *Q* and $Q^{\prime }$ under the uniform profile; $\pi_{i}=1/20$. Under this transformation $Q_{ij}\propto S_{ij}$ is a function of *i* and *j*, so the rate matrix entries can be thought of as exchangeabilities and we refer to them as such. But the transformation to *Q* and $Q^{\prime }$ puts the exchangeabilities onto a more comparable scale, one that is more closely associated with their end-use in rate matrices than setting one entry to 1 as was done in optimization.

## Data Sets

Because of the computational burden associated with the estimation of the exchangeabilities in the GTRpmix model, we have analyzed two large concatenated protein “super-matrix” datasets to estimate “general use” substitution matrices for phylogenetic estimation with profile mixture models. These can be used with profile mixture models when sufficient computational resources are not available for full GTRpmix optimization or the datasets to be analyzed are too small to allow accurate estimation. The two datasets used to estimate these matrices are a pan-eukaryotic concatenated supermatrix and a eukaryote-archaea supermatrix:

**Pan-Eukaryotic data sets**: To estimate the “Eukaryote” exchangeability matrix we selected a 240-protein data set with 76,840 sites and 78 taxa as a taxonomically representative subsample of all eukaryotes in the PhyloFisher database (Tice et al. 2021). Taxa were selected based on their known membership in a particular higher-level eukaryotic taxon and their phylogenetic position. Further selection was done to maximize gene coverage within the original PhyloFisher data set. As detailed below, to compare two methods of exchangeability estimation, we also looked at a smaller subset of the PhyloFisher database consisting of a 240-protein data set with 77,965 sites 50 taxa.

**Eukaryotic-Archaeal data set**: To estimate the exchangeability matrix for reconstructing relationships between eukaryotes and Archaea, we used a 54-protein data set with 14,704 sites and 86 taxa. This data set includes a subset of the taxa presented in Eme et al. (2023).

For more details on the datasets and their taxonomic selection, see the Supplement’s section “*Data Sets*.”

### Data Sets for Comparisons

The following data set is used to compare the fit of the new empirically estimated matrices to be used for reconstructing relationships between eukaryotes and Archaea against the LG matrix and the one estimated for Eukaryotic phylogenetic analysis.

- a data set of 56 ribosomal proteins (7,112 sites × 86 taxa) described in Eme et al. (2023). To ensure computational tractability taxa were subsampled from the original 331 taxon dataset to maintain a representation of Asgard archaea, TACK archaea, and Euryarchaeota. A tree topology, denoted $T_{R}$ estimated under LG+C60+G4, is used for comparing different exchangeabilities matrices.

We also used three empirical concatenated super-matrices to validate the empirical-estimated matrix discussed above to be used for Eukaryotic phylogenetic analysis and compare it with the LG matrix. For each data set, we consider two trees, the correct topology and an artifactual one (product of long-branch attraction artifacts). The data sets and trees are as follows:

- a data set of the 133-protein data set (24,291 sites × 40 taxa) described in (Brinkmann et al. 2005) to assess the placement of the Microsporidia in the tree of eukaryotes. The correct topology denoted $T_{M}$, was originally recovered with LG+C20+F+G4, (Susko et al. 2018) and places the Microsporidia branch as sister to Fungi. The artifactual topology denoted $T_{\mathrm{MA}}$, was recovered with LG+F+G4 and groups the Microsporidia with the archaeal outgroup (i.e. branching sister to all other eukaryotes) due to an LBA artifact.
- a data set of 146 proteins (35,371 sites × 37 taxa) described in Lartillot et al. (2007) to assess the placement of the Nematodes in the animal tree of life. The correct topology, denoted $T_{N}$, was recovered with LG+C20+F+G4 where Nematodes branch as sister to arthropods. The artifactual topology denoted $T_{\mathrm{NA}}$, was recovered with LG+F+G.
- a data set of 146 proteins (35,371 sites × 32 taxa) assembled in Lartillot et al. (2007) to assess the position of the Platyhelminths in the animal tree of life. The correct topology denoted $T_{P}$, was recovered with CAT+GTR places Platyhelminths within the Protostomia. The artifactual topology, denoted $T_{\mathrm{PA}}$, recovered with LG+F+G4 and places Platyhelminths within Coelomata and many mixture models (see Lartillot et al. 2007; Wang et al. 2017; Susko et al. 2018)

## Parameter Estimation Performance

To validate our implementation, we simulated 100 MSAs, with 10,000 sites and 10 taxa, using Alisim (Ly-Trong et al. 2022). Each alignment was simulated under the following conditions: LG exchangeabilities; a profile mixture model with four profiles, we arbitrarily chose the first four profiles from the C60 model, which turn out to be quite distinct (supplementary fig. S2, Supplementary Material online); a 10-taxon tree, depicted in supplementary fig. S1, Supplementary Material online, obtained from the empirically estimated tree $T_{M}$ defined above, after randomly removing taxa; and a discretized gamma distribution G4 (Yang 1994), with α=0.67, where *α* was chosen from an empirical data estimate (obtained after fitting the model LG+C60+G4 on the tree $T_{M}$ for the Microsporidia data set). The arbitrarily chosen weights of the profiles were 0.35, 0.15, 0.25, and 0.25, respectively. For each MSA, we jointly estimated exchangeabilities, branch lengths, profile weights, and the rate parameter *α*. The only parameters not estimated were the tree topology and the profiles. We chose the POISSON exchangeability matrix, where all entries are equal to 1, as the initial values for the exchangeabilities to guarantee that the success of optimization was not due to the starting values being close to the true values.

**Fig. 1.**
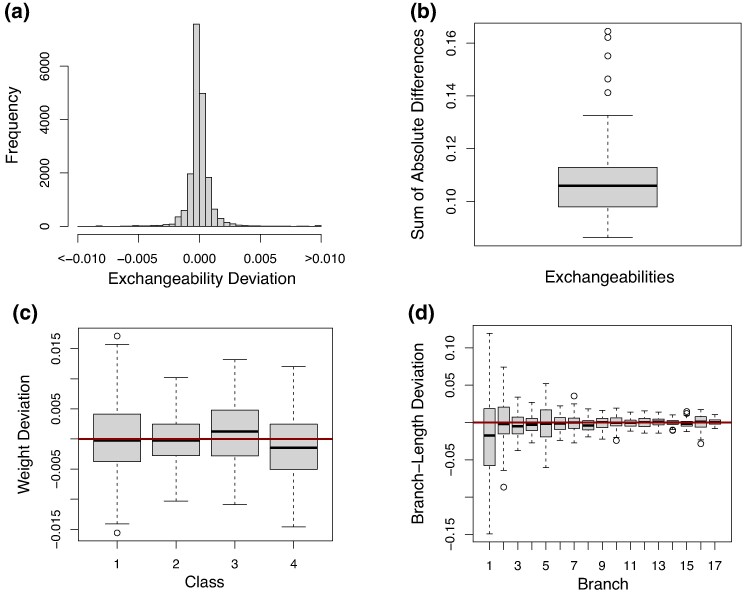
Plots showing the comparison between true and estimated parameters for all 100 simulations. For the box plots, an horizontal line at zero represents a perfect estimation of the true parameters. a) Histogram showing the differences between true and estimated exchangeability deviation for all entries. b) Box plot showing the sum of absolute differences between the true (LG) and estimated exchangeabilities. c) Box plot showing the differences between true and estimated weights for the four classes used to simulate the data. d) Box plot showing the differences between true and estimated branch lengths. Branches are ordered from largest to shortest (the reason why variability decreases from left to right).

Figure 1a shows a histogram of the difference between true and estimated exchangeability deviation for all entries and for all 100 simulations. Particularly, this plot shows how most entries were accurately inferred since most of the mass is around zero. Supplementary fig. S3, Supplementary Material online gives separate box plots for each exchangeability entry and shows that all entries are adequately estimated.

**Fig. 2.**
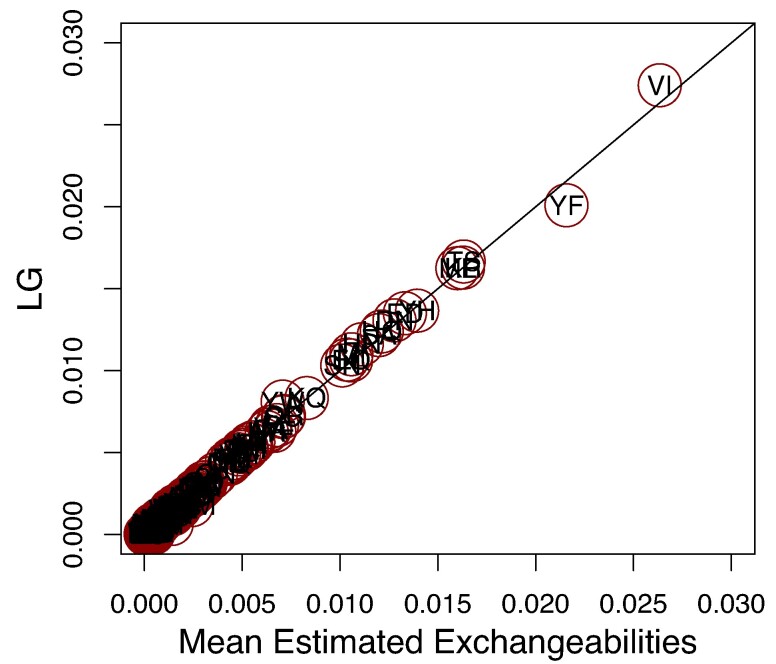
Entry-wise comparison between the mean exchangeabilities estimated from the simulated data and the LG exchangeabilities used to generate the data. Each dot represents an entry in the exchangeability matrix, where the *x*-coordinate is the mean estimated exchangeability over all 100 simulations and the *y*-coordinate is the corresponding LG entry. Each point is labeled with the two amino acids it represents. All circles have equal sizes and were chosen to fit the label of the exchangeability they represent.

To investigate the performance of estimation of all exchangeabilities jointly, we compute for each estimated matrix *S*, the sum of absolute differences (SAD) between the true exchangeability matrix (LG) and *S*. Figure 1b shows the box plot of the SAD for all simulations. For reference, the SAD between the true matrix (LG) and the starting matrix (POISSON) is ∼0.5, which is considerably larger than that of any estimated matrices. Moreover, the SAD between the LG matrix, and the mean estimated matrix is ∼0.02 (Fig. 2), suggesting consistency of the exchangeability estimation.

Other parameters optimized jointly with exchangeabilities were also accurately estimated including profile weights (Fig. 1c), branch lengths (Fig. 1d), and the alpha shape parameters (supplementary fig. S4, Supplementary Material online).

Over 100 simulations, the median total CPU time used to estimate all parameters for each simulated dataset is 4,297 s and the median wall-clock is 217 s on an Intel Xeon E5-2697 with 64 GB RAM when using IQ-TREE2’s multithreading option on 20 cores.

## Estimating Exchangeabilities Improves Topological Accuracy

In Baños et al. (2024), it was shown that misspecification of the exchangeabilities can severely hamper tree estimation. Specifically, it was shown that, under a “rich” profile mixture model, data simulated under POISSON exchangeabilities and fitted using fixed LG exchangeabilities together with a profile mixture model that includes an “F-class” (a profile that is defined from the empirical frequencies of amino acids from the MSA) performs poorly.

To determine if GTR estimation would address this problem, we investigated a similar scenario by simulating 100 MSAs of length 10,000 using the POISSON+C10+G4{0.5} model on a 12-taxon tree shown in supplementary fig. S5, Supplementary Material online (L). We separately fitted the profile mixture model C10+F+G4{0.5} using the POISSON, LG, and GTR exchangeabilities, and two tree topologies, the correct tree and an artifactual one corresponding to the long-branch attraction (LBA) artifact (supplementary fig. S5, Supplementary Material online (R)). For all models, branch lengths and weights of the profiles were estimated, and for GTR+C10+F+G4{0.5} exchangeabilities were also estimated. Table 1 shows, for each model, the proportion of times the true tree had a higher log-likelihood than the LBA tree. As expected (Baños et al. 2024), LG+C10+F performs poorly when compared to the POISSON+C10+F model; GTR+C10+F, performs much better than LG and is much closer in performance to the POISSON+C10+F model. If more taxa and sites were considered, the GTR exchangeability estimates are expected to approach the true POISSON exchangeabilities and tree estimation would improve concomitantly. Note that if profiles were misspecified, we would expect heterogeneity in the estimated exchangeabilities that compensate for this even with a very large data set or more taxa.

**Table 1:** Proportion of times the correct tree is preferred over the artifactual LBA tree for 100 simulated MSAs of length n=500 and n=10,000, simulated under the model POISSON+C10+G4{0.5}

	Proportion correct	
Model	n=500	n=10,000
POISSON+C10+F+G4{0.5}	0.64	0.96
LG+C10+F+G4{0.5}	0.46	0.58
GTR+C10+F+G4{0.5}	0.64	0.83

The only difference between fitted models is the choice of exchangeabilities. For the model GTR+C10+F+G4{0.5}, exchangeabilities are estimated using our implementation. McNemar’s test of the equality of proportions yielded a *P*-value ∼0 when comparing the contingency table from trees for models GTR+C10+F+G4{0.5} and LG+C10+F+G4{0.5} for the MSAs with 10,000 sites.

Table 1 also shows a similar scenario to the one described above, with the only difference being that all MSAs are of length 500 sites. We note that for both the true tree and the LBA tree, for the MSA’s of length 10,000, the mean SAD between the true POISSON and the estimated GTR exchangeabilities is ∼0.27, while for the MSAs of length 500 SAD is ∼0.9. Comparing the results from both MSA’s lengths, we see that the proportions of correct topological estimates increase much less for the incorrect LG-based model than for either the POISSON or GTR-based models. It is also noteworthy that the POISSON and GTR proportions obtained from the MSAs of length 500 are comparable despite POISSON requiring far fewer parameters to be estimated. We strongly suspect that this is the result of a small sample bias as described in Wang et al. (2019).

## Data Analysis

We then investigated if GTRpmix improves model fit significantly compared to the LG matrix for the Microsporidia data set from Brinkmann et al. (2005). By fixing the tree topology $T_{M}$ and the profiles of model C60, we jointly estimated the exchangeabilities of the GTR model, class weights, *α* from a discretized G4, and branch lengths. We refer to the estimated exchangeability matrix in this section as the Miscrosporidia eXchangeability Matrix (MXM). We compare this model against LG+C60+G4, where class weights, *α*, and branch lengths are estimated under fixed tree topology $T_{M}$, profiles of model C60, and LG exchangeabilities. Table 2 shows the log-likelihoods, AIC, and BIC obtained from models LG+C60+G4 and MXM+C60+G4. Note that for MXM+C60+G4, 189 additional parameters are being estimated compared to LG+C60+G4. Nevertheless, the AIC and BIC scores suggest a preference for exchangeability estimation by a large margin (around 10k AIC and BIC units, Table 2).

**Table 2:** The log-likelihoods, number of free parameters (denoted *k*, which also accounts for the 77 branch lengths in the tree), AIC, and BIC obtained from fitting models MXM+C60+G4, LG+C60+G4, FM+F+G4, and FM+C60+G4

	LH	*k*	AIC	BIC
MXM+C60+G4	− **710,812**	326	**1,422,276**	**1,424,916**
LG+C60+G4	− 716,579	137	1,433,432	1,434,541
FM+F+G4	− 727,849	286	1,456,270	1,458,586
FM+C60+G4	− 715,659	326	1,431,970	1,434,610

The best values per column are shown in bold. Both exchangeability matrices MXM and FM were estimated from the data, with the former estimated under C60 and the latter with the single frequency class (denoted by “+F” in IQ-TREE2) based on the frequencies of amino acids in the alignment.

**Fig. 3.**
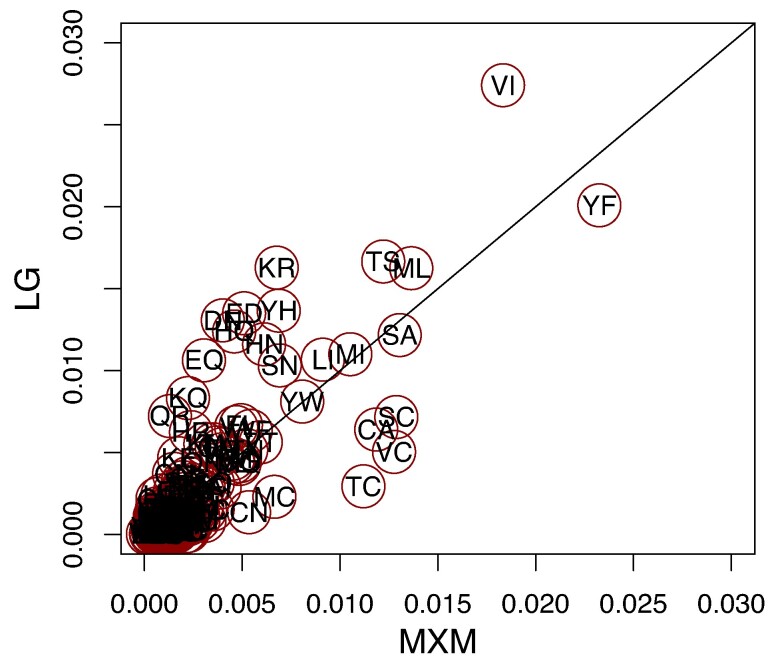
Entry-wise comparison between the MXM matrix, obtained from fitting a GTR matrix to the Microsporidia data set, and the LG exchangeabilities. Each dot represents an entry in the exchangeability matrix, where the *x*-coordinate is the entry of the MXM matrix and the *y*-coordinate is its corresponding LG matrix entry. Each point is labeled with the two amino acids it represents. All circles have equal sizes and were chosen to fit the label of the exchangeability they represent.

Figure 3 shows a comparison between the entries of the LG and the MXM matrix. Particularly, we see that many of the entries in the LG matrix are different than in the MXM matrix. Note that the SAD between POISSON and MXM exchangeabilities is ∼0.36 and between LG and POISSON is ∼0.5. To provide insight into these differences, we focus on a particular example. Some exchangeabilities involving cysteine (C) are increased in MXM compared to LG. This is likely because in C60 there is a profile (profile 8 as listed in IQ-TREE2) where cysteine has a frequency of ∼0.42 and both alanine and serine each have a frequency of ∼0.18; these three amino acids account for almost 80% of the overall amino acid proportion of this profile. By excluding from C60 this and profile 4 (where cysteine has a frequency of 0.16 and only A, L, S, T, and V have a frequency greater than 0.05), the mean frequency of cysteine in the 58 remaining profiles is ∼0.009. Assuming this profile mixture is closer to reality, even if the exchangeabilities involving cysteine are non-negligible, a frequency profile with a substantial probability of cysteine is unlikely. Thus, a small proportion of sites with a cysteine and some other amino acid is expected. Because MXM takes frequency profiles into account it can recognize this. However, when a single profile is assumed in fitting, as with LG, a small exchangeability provides the only way to account for a low frequency of sites with cysteine and some other amino acids. A bubble plot of the differences between the LG and MXM exchangeabilities can be found in supplementary fig. S6, Supplementary Material online.

For comparison, we also estimated a GTR matrix, denoted FM, under a single profile and discretized G4 rates for the Microsporidia data set. Specifically, for the profile we used the overall frequencies of amino acids in the data set. Supplementary fig. S7, Supplementary Material online(L) depicts a comparison between the FM and MXM matrices. This figure shows, as expected, how MXM has higher cysteine exchangeabilities than FM, as was noted in the MXM to LG comparison. For additional comparison, the SAD between the FM and LG is ∼0.15, while the SAD between the FM and MXM matrices is ∼0.26. This means that the FM matrix is more similar to the LG matrix than the MXM matrix. Table 2 shows that the AIC, and BIC scores of FM+F+G4, LG+C60+G4, and FM+C60+G4 are all worse than MXM underscoring the importance of fitting multiple profiles and exchangeabilities jointly. As suggested in Minh et al. (2021) and Pandey and Braun (2020), different clades are likely to have different optimal models, and thus one would expect the model FM+C60+G4 to perform better than the model LG+C60+G4. While in fact, the FM+C60+G4 model is favored over LG+C60+G4 according to the AIC it is not according to the BIC. Because AIC and BIC were derived assuming full ML estimation of all parameters but FM+C60+G4 was not estimated in this way, it is not clear that these are the right criteria for comparison. BIC, in particular, was derived to approximate Bayes factors for models and usually assumes that ML estimates are used in calculating likelihoods (Schwarz 1978).

The total running time for full estimation of the exchangeabilities for the MXM model, branch lengths, C60 class weights, and the shape parameter, *α* was ∼11 hours on 40 cores of an AMD EPYC 7,543 Processor with 2T of RAM. We investigated whether accurate estimation of exchangeabilities would be possible by fixing branch lengths and the shape parameter *α* from G4. Specifically, we estimated the exchangeability matrix, denoted MXM^*Fix*^, by fixing branch lengths and *α* to the averages of these parameters obtained from fitting POISSON+C60+G4 and LG+C60+G4. The total running time to estimate MXM^*Fix*^ was ∼7 hours on the same computer and the resulting SAD between MXM and MXM^*Fix*^ is 0.017, suggesting the estimates are very similar (supplementary fig. S7, Supplementary Material online(R) shows an entry-wise comparison between these two matrices). The log-likelihood obtained after fitting the model MXM^*Fix*^+C60+G4 (where branch lengths and *α* are re-estimated) is only ∼73 likelihood units less than the one obtained from fitting MXM+C60+G4 with all parameters estimated simultaneously. This suggests that fixing branch lengths and *α* does not greatly affect the estimation of exchangeabilities while yielding important computation time savings.

## Empirically Estimated Exchangeability Matrices

Since the estimation of exchangeabilities is computationally expensive even after fixing branch lengths and rates, many users will not have the computational resources to optimize matrices for their datasets of interest. Alternatively, users may have datasets that are not large enough to permit accurate exchangeability estimation (e.g. a single-protein alignment). For these reasons, we have estimated two exchangeability matrices from large data sets using the C60+G4 model that can be used as fixed matrices for phylogenetic analyses under profile mixture models. The first matrix we introduce is tailored for phylogenetic analyses of proteins encoded by eukaryotic nuclear genes and the other is for reconstructing relationships between nuclear-encoded proteins in eukaryotes and orthologs in Archaea.

### The ELM Exchangeability Matrix for Eukaryotic Analyses

We estimated an exchangeability matrix, which we refer to as the **E**ukaryotic **L**inked **M**ixture (ELM) matrix. This matrix was estimated from the 78-taxon Pan-Eukaryotic data set described in the section “*Data Sets*” above. We used the profiles from model C60, discretized G4 rates, and a tree topology recovered by fitting LG+C60+G4 to the data depicted in supplementary fig. S10, Supplementary Material online. To reduce computational time, we used the approach described for MXM^*fix*^ estimation above; i.e. we fixed branch lengths and *α* to their averages based on estimates from LG+C60+G4 and POISSON+C60+G4. Thus for the ELM matrix estimation, we only optimized exchangeabilities and C60 profile weights jointly.

**Fig. 4.**
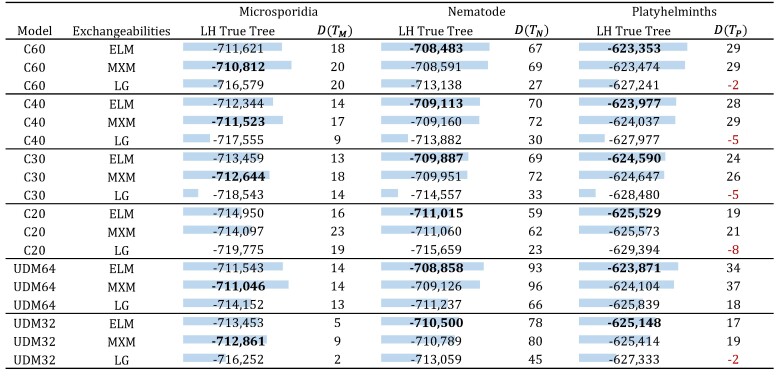
The log-likelihoods of the trees estimated from the three empirical data sets and the difference between fitting the true and the artifactual tree for each data set. The bar scale goes from the lower value per column (empty) to the highest value (full) per column. $D(T_{X})$ denotes the log-likelihood of the “correct tree” (e.g. $T_{M}$) minus the “incorrect” tree (e.g. $T_{\mathrm{MA}}$). Positive values of $D(T_{X})$ reflect a preference for the true tree, while negative preference for the LBA tree. The best LH score per model and data set is shown in bold. In the first column, the MXM matrix is the GTR matrix for the data set represented in the column (Microsporidia dataset), showing that the ELM matrix gives similar log-likehoods to the GTR exchangeability rates.

Supplementary fig. S8, Supplementary Material online shows a bubble plot of the difference between the LG and ELM exchangeabilities, and supplementary fig. S9, Supplementary Material online(L) depicts an analog of Fig. 3 for these two matrices. To compare the fit between these two matrices, we used the three empirical data sets (Microsporidia, Nematode, and Platyhelminths) described in the subsection “*Data Sets for Comparisons*.” Figure 4 contains, among other things, the likelihoods obtained from fitting models LG+C60+G4 and ELM+C60+G4 for the correct and artifactual topologies of each data set. Note that, by applying the KHns test developed in Susko (2014) to compare two fixed tree topologies, a likelihood difference between fitting the true and artifactual tree is considered significant, with a 5% significance level, if it is greater than 5.53 for the Microsporidia, 2.99 for the Nematode, and 1.92 for the Platyhelminths data sets. Although the KHns test does not correct for the selection bias induced by estimation of the artifactual tree from the data, nevertheless, these thresholds do give some indication about how large likelihoods might be expected to be under the null hypothesis. Clearly, the ELM matrix produces considerably better likelihood scores for all data sets than the LG matrix. We note that for the Platyhelminths data set the ELM+C60+G4 significantly prefers the true tree over the artefactual tree, whereas the LG+C60+G4 matrix does not. For the other two datasets, all models preferred the true tree, although the LG model consistently had the weakest preference, Fig. 4.

We also found that even when the ELM matrix was optimized using the profiles from C60, it still provided better fit and improved topological accuracy when fitting different sets of profiles. Figure 4 contains the likelihoods for the three data sets when fitting the profiles in C40, C30, C20, UDM32, and UDM64 with LG and ELM exchangeabilities. For all profile mixture models, the ELM matrix provides better likelihood scores than the LG matrix. We also see that the ELM matrix always prefers the true tree, which is not true for the LG matrix under models C20, C40, and UDM32 for the Platyhelminths data set.

To make a broader comparison, we also looked at the likelihoods for all the profile mixture models used in the comparisons above with MXM exchangeabilities, shown in Fig. 4. As expected, independently of the profiles, for the Microsporidia data set the MXM matrix produces better likelihood scores since this matrix was optimized on this data set. Nonetheless, for the other two data sets the ELM matrix produces better likelihood scores.

To confirm that the choice of branch lengths and *α* did not negatively affect the estimation of the ELM matrix, we also estimated an exchangeability matrix with the C60+G4 model, referred to as ELM50, on the 50-taxon Pan-Eukaryotic matrix described in the “*Data Sets*” section. We chose this smaller data set to ease some of the computational burden so that joint exchangeability, profile weight, branch length, and *α* estimation could be conducted. The entry-wise comparison between the ELM and ELM50 matrices in supplementary fig. S9, Supplementary Material online(R) shows that these two matrices are in general very similar (the SAD between these matrices is ∼0.034). Supplementary table S1, Supplementary Material online shows the equivalent of Fig. 4 for the ELM50 matrix. We note that in all cases the ELM matrix produces better likelihood scores than the ELM50 matrix. We conclude that estimating exchangeabilities using fixed branch lengths and *α* did not affect negatively the estimation of the ELM matrix. Using fixed parameters may allow many more taxa to be used which should improve estimation. However, in cases where the guide tree and parameters, are far from optimal, it could lead to poor estimation.

**Fig. 5.**
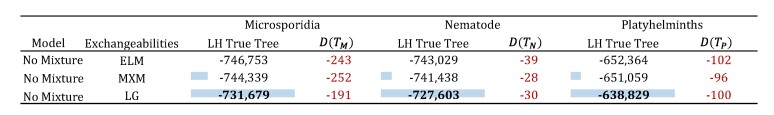
Similar to Fig. 4 but for models with a single profile, denoted as F, estimated from the overall frequencies of amino acids in each data set.

On the other hand, the performance of the ELM matrix under a single profile model is subpar compared to the LG matrix (Fig. 5). With a single “F-class” for each data set, both ELM and MXM perform worse than LG, so it is inadvisable to use these models as part of a site-homogeneous model with a single profile. Although LG performs better than the other matrices for this site-homogeneous setting, it does considerably worse than these matrices when used with any of the mixture models in Fig. 4.

### The EAL Exchangeability Matrix for Reconstructing Relationships between Eukaryotes and Archaea

We estimated an exchangeability matrix, which we refer to as the **E**ukaryotic and **A**rcheal **L**inked mixture (EAL) matrix, from the 86-taxon Eukaryotic-Archaeal data set described in the section “*Data Sets*” above. We used the profiles from model C60, discretized G4 rates, and the tree topology depicted in supplementary fig. S10.5, Supplementary Material online, which is assumed to be the correct one. Similar to how we estimated the matrix MXM^*fix*^, we fixed branch lengths and *α* to their average from fitting models LG+C60+G4 and POISSON+C60+G4.

Supplementary figs. S11 and S12, Supplementary Material online show a comparison of the EAL, ELM, and LG matrices. These plots indicate that these matrices are all quite different, with the EAL matrix being somewhat more similar to ELM than to LG. The SAD from ELM and EAL is ∼0.19, whereas the SAD between LG and EAL is ∼0.26.

To show this matrix gives a better fit for data sets with both eukaryotic and archaeal sequences, we used the 56 ribosomal protein data set and the tree $T_{R}$ described in the *Data Sets* section. Table 3 shows the log-likelihood obtained by fitting models LG+C60+G4, ELM+C60+G4, and EAL+C60+G4 for this data set and tree. It is clear that EAL produces the best likelihood score by a wide margin. Note that since all the models have the same number of parameters, their fit can be directly compared using the log-likelihood scores (under these conditions, AIC and BIC will yield identical orderings of relative model fit to log-likelihood comparisons).

**Table 3:** The log-likelihoods obtained from fitting models LG+C60+G4, ELM+C60+G4, and EAL+C60+G4 on the for the 56 ribosomal protein data set and the tree $T_{R}$

Model	LH
LG+C60+G4	− 745,005
ELM+C60+G4	− 738,773
EAL+C60+G4	− **736,404**

The highest log-likelihood value is shown in bold.

## Conclusion

We have shown that estimation of linked exchangeabilities jointly with profile mixture model weights in the GTRpmix model framework provides substantially better model fit for empirical amino acid alignments, and it can improve topological estimation for especially difficult problems where widely used empirical exchangeability matrices like LG fail. The GTRpmix model will be extremely useful for researchers investigating deep phylogenetic problems where the use of well-fitting site-heterogeneous models is especially important to avoid phylogenetic artifacts. Furthermore, we provide a number of pre-estimated matrices for use with profile mixture models in the analysis of eukaryotic nucleus-encoded protein data sets (e.g. MXM, ELM) and eukaryote-archaeal data sets (e.g. EAL).

Matrices ELM and EAL are available to use in the IQ-TREE2 software version v2.3.1.

## Supplementary Material

msae174_Supplementary_Data

## Data Availability

The data underlying this article, together with the ELM, EAL, MXM and ELM50 matrices, are also available in the Github repository https://github.com/RogerLab/GTRPMIX/.

## Supplementary Material

Supplementary material is available at *Molecular Biology and Evolution* online. Additionally, in the Supplementary material online, we have included an IQ-TREE2 sample command line to estimate exchange abilities as it was done in this work.

## References

[msae174-B1] Adachi J, Waddell PJ, Martin W, Hasegawa M. Plastid genome phylogeny and a model of amino acid substitution for proteins encoded by chloroplast DNA. J Mol Evol. 2000:50(4):348–358. 10.1007/s002399910038. 10795826

[msae174-B2] Baños H, Susko E, Roger AJ. Is over-parameterization a problem for profile mixture models? Syst Biol. 2024:73(1):53–75. 10.1093/sysbio/syad063. 37843172 PMC11129589

[msae174-B3] Brinkmann H, van der Giezen M, Zhou Y, de Raucourt GP, Philippe H. An empirical assessment of long-branch attraction artefacts in deep eukaryotic phylogenomics. Syst Biol. 2005:54(5):743–757. 10.1080/10635150500234609. 16243762

[msae174-B4] Dayhoff M, Schwartz R, Orcutt B. A model of evolutionary change in proteins. In: Dayhoff M, editor. Atlas of protein sequence and structure. vol. 5. Washington, D.C.: National Biomedical Research Foundation; 1978. p. 345–352.

[msae174-B5] Dimmic MW, Rest JS, Mindell DP, Goldstein RA. rtREV: an amino acid substitution matrix for inference of retrovirus and reverse transcriptase phylogeny. J Mol Evol. 2002:55(1):65–73. 10.1007/s00239-001-2304-y. 12165843

[msae174-B6] Eme L, Tamarit D, Caceres EF, Stairs CW, De Anda V, Schön ME, Seitz KW, Dombrowski N, Lewis WH, Homa F, et al. Inference and reconstruction of the heimdallarchaeial ancestry of eukaryotes. Nature. 2023:618(7967):992–999. 10.1038/s41586-023-06186-2. 37316666 PMC10307638

[msae174-B7] Fletcher R . Practical methods of optimization. 2nd ed. New York: John Wiley & Sons, Ltd; 1987.

[msae174-B8] Franzosa EA, Xia Y. Structural determinants of protein evolution are context-sensitive at the residue level. Mol Biol Evol. 2009:26(10):2387–2395. 10.1093/molbev/msp146. 19597162

[msae174-B9] Goldstein RA . The structure of protein evolution and the evolution of protein structure. Curr Opin Struct Biol. 2008:18(2):170–177. 10.1016/j.sbi.2008.01.006. 18328690

[msae174-B10] Jones DT, Taylor WR, Thornton JM. The rapid generation of mutation data matrices from protein sequences. Bioinformatics. 1992:8(3):275–282. 10.1093/bioinformatics/8.3.275. 1633570

[msae174-B11] Lartillot N, Brinkmann H, Philippe H. Suppression of long-branch attraction artefacts in the animal phylogeny using a site-heterogeneous model. BMC Evol Biol. 2007:7(1):S4. 10.1186/1471-2148-7-S1-S4. PMC1796613 17288577

[msae174-B12] Lartillot N, Philippe H. A Bayesian mixture model for across-site heterogeneities in the amino-acid replacement process. Mol Biol Evol. 2004:21(6):1095–1109. 10.1093/molbev/msh112. 15014145

[msae174-B13] Lartillot N, Rodrigue N, Stubbs D, Richer J. PhyloBayes MPI: phylogenetic reconstruction with infinite mixtures of profiles in a parallel environment. Syst Biol. 2013:62(4):611–615. 10.1093/sysbio/syt022. 23564032

[msae174-B14] Le SQ, Gascuel O. An improved general amino acid replacement matrix. Mol Biol Evol. 2008:25(7):1307–1320. 10.1093/molbev/msn067. 18367465

[msae174-B15] Ly-Trong N, Naser-Khdour S, Lanfear R, Minh BQ. AliSim: a fast and versatile phylogenetic sequence simulator for the genomic era. Mol Biol Evol. 2022:39(5):msac092. 10.1093/molbev/msac092. 35511713 PMC9113491

[msae174-B16] Minh BQ, Dang CC, Vinh LS, Lanfear R. QMaker: fast and accurate method to estimate empirical models of protein evolution. Syst Biol. 2021:70(5):1046–1060. 10.1093/sysbio/syab010. 33616668 PMC8357343

[msae174-B17] Pál C, Papp B, Lercher MJ. An integrated view of protein evolution. Nat Rev Genet. 2006:7(5):337–348. 10.1038/nrg1838. 16619049

[msae174-B18] Pandey A, Braun EL. Protein evolution is structure dependent and non-homogeneous across the tree of life. In: Proceedings of the 11th ACM International Conference on Bioinformatics, Computational Biology and Health Informatics, BCB ’20. New York (NY), USA: Association for Computing Machinery; 2020.

[msae174-B19] Schrempf D, Lartillot N, Szöllősi G. Scalable empirical mixture models that account for across-site compositional heterogeneity. Mol Biol Evol. 2020:37(12):3616–3631. 10.1093/molbev/msaa145. 32877529 PMC7743758

[msae174-B20] Schwarz G . Estimating the dimension of a model. Ann Stat. 1978:6(2):461–464. 10.1214/aos/1176344136.

[msae174-B21] Si Quang L, Gascuel O, Lartillot N. Empirical profile mixture models for phylogenetic reconstruction. Bioinformatics. 2008:24(20):2317–2323. 10.1093/bioinformatics/btn445. 18718941

[msae174-B22] Spielman SJ, Wilke CO. The relationship between dN/dS and scaled selection coefficients. Mol Biol Evol. 2015:32(4):1097–1108. 10.1093/molbev/msv003. 25576365 PMC4379412

[msae174-B23] Susko E . Tests for two trees using likelihood methods. Mol Biol Evol. 2014:31(4):1029–1039. 10.1093/molbev/msu039. 24401182

[msae174-B24] Susko E, Lincker L, Roger AJ. Accelerated estimation of frequency classes in site-heterogeneous profile mixture models. Mol Biol Evol. 2018:35(5):1266–1283. 10.1093/molbev/msy026. 29688541

[msae174-B25] Tavaré S, Miura RM. Some probabilistic and statistical problems on the analysis of DNA sequences. Lectures Math Life Sci. 1986:17:57–86.

[msae174-B26] Tice AK, Zihala D, Panek T, Jones RE, Salomaki ED, Nenarokov S, Burki F, Elias M, Eme L, Roger AJ, et al. PhyloFisher: a phylogenomic package for resolving eukaryotic relationships. PLoS Biol. 2021:19(8):1–22. 10.1371/journal.pbio.3001365. PMC8345874 34358228

[msae174-B27] Wang H-C, Li K, Susko E, Roger AJ. A class frequency mixture model that adjusts for site-specific amino acid frequencies and improves inference of protein phylogeny. BMC Evol Biol. 2008:8(1):331. 10.1186/1471-2148-8-331. 19087270 PMC2628903

[msae174-B28] Wang H-C, Minh BQ, Susko E, Roger AJ. Modeling site heterogeneity with posterior mean site frequency profiles accelerates accurate phylogenomic estimation. Syst Biol. 2017:67(2):216–235. 10.1093/sysbio/syx068. 28950365

[msae174-B29] Wang H-C, Susko E, Roger AJ. An amino acid substitution-selection model adjusts residue fitness to improve phylogenetic estimation. Mol Biol Evol. 2014:31(4):779–792. 10.1093/molbev/msu044. 24441033

[msae174-B30] Wang H-C, Susko E, Roger AJ. The relative importance of modeling site pattern heterogeneity versus partition-wise heterotachy in phylogenomic inference. Syst Biol. 2019:68(6):1003–1019. 10.1093/sysbio/syz021. 31140564

[msae174-B31] Whelan S, Goldman N. A general empirical model of protein evolution derived from multiple protein families using a maximum-likelihood approach. Mol Biol Evol. 2001:18(5):691–699. 10.1093/oxfordjournals.molbev.a003851. 11319253

[msae174-B32] Wong TKF, Cherryh C, Rodrigo AG, Hahn MW, Minh BQ, Lanfear R. MAST: phylogenetic inference with mixtures across sites and trees. Syst Biol. 2024:73(2):375–391. 10.1093/sysbio/syae008. 38421146 PMC11282360

[msae174-B33] Yang Z . Maximum likelihood phylogenetic estimation from DNA sequences with variable rates over sites: approximate methods. J Mol Evol. 1994:39(3):306–314. 10.1007/BF00160154. 7932792

